# Innovation and Challenges in Materials for Transcatheter Aortic Valves

**DOI:** 10.1007/s12265-026-10840-1

**Published:** 2026-09-05

**Authors:** Pramod Dorishetty, Aden Carlton, Leon Neethling, Filippo Valente

**Affiliations:** 1 Ear Science Institute Australia, Perth, Australia; 2 Centre for Ear Science, School of Medicine, The University of Western Australia, Perth, Australia; 3 Anteris Technologies Ltd, Perth, Australia

**Keywords:** Aortic stenosis, Heart-valves, Trans-catheter aortic valve implantation (TAVI), Biomaterials

## Abstract

Transcatheter Aortic valve implantation (TAVI) procedure has been revolutionizing the Aortic Stenosis (AS) treatment over the last 20 years, because of its minimally invasive approach and fast postoperative recovery. However, innovation in valve material compatible with TAVI procedure present significant challenges, as they must endure the demanding conditions of catheter-based delivery. This review outlines the fundamental material requirements and challenges in the design and manufacturing of TAVI valves. It compares the existing TAVI devices made from animal tissues and polymers, and identifies the key factors that are limiting their adoption for widespread clinical use. While biological valves remain the predominant choice in current clinical TAVI practice, major future innovation is likely to arise from advances in polymer science aimed at overcoming the key limitations like calcification and limited durability. Fully polymeric valve systems are still at an early stage of development, but they hold considerable long-term potential due to their tunable mechanical properties, resistance to calcification, and potential for scalable manufacturing, that could revolutionize the field.

**Graphical Abstract:**

**Figure:**
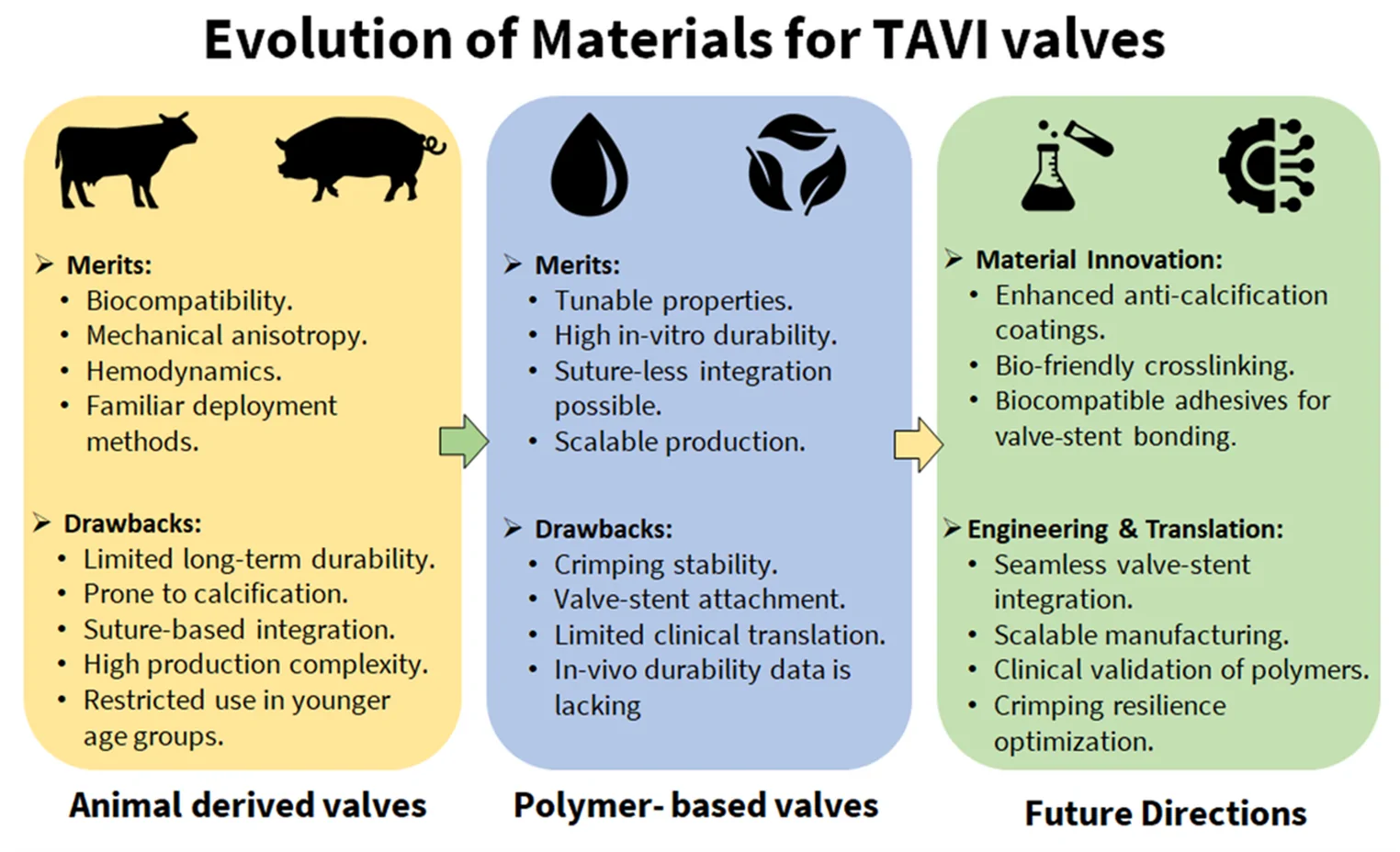
ToC Figure

## Introduction

Aortic Stenosis (AS) is the most common heart valve disease affecting nearly 9 million people globally, with its prevalence increasing rapidly due to increase in aging population [1]. Around 2–3% of individuals aged over 65 years have AS, which is characterized as thickening of the aortic valve leaflets that restricts the optimal blood flow, causing shortness of breath (dyspnea), heart rhythm problems and death in severe cases [2]. More than 180,000 heart valve replacements are performed in the United States every year with the demand for artificial heart valves increasing at a rate of 10–12% per year [3]. It is forecasted that the total number of heart valve replacements by 2050 will be approximately 850,000 per year [4]. The increase in demand drives the research and development towards scalable novel solutions to meet the demand and perform the heart-valve replacements safely and effectively.

Over the last 70 years, materials used to fabricate artificial heart valves have evolved significantly, transitioning from purely mechanical to bioprosthetic tissues and polymeric heart valves [5]. Mechanical heart-valves were the first-generation valves made of steel and other alloys, offering high mechanical strength and long-term stability. However, they are prone to thrombosis which requires a life-long anti-coagulation treatment [6]. Moreover, their implantation requires open heart surgery, hence often not an option for high-risk patients including aged population and people with existing comorbidities [7]. Bioprosthetic heart valves, introduced in early 1970s, are made of chemically treated animal tissue (mostly bovine or porcine pericardium) [8, 9]. These valves offer better flexibility when compared to mechanical heart valves and do not require long-term treatments. However, they exhibit limited durability due to leaflet calcification and degeneration [10]. Polymeric heart valves represent the next generation heart valves, made of naturally-derived, or synthetic polymers, or a combination thereof. Both bioprosthetic and polymeric valves can be implanted using minimally invasive procedures, which makes them more suitable candidates for the high-risk patients that cannot undergo open-heart surgery.

**Fig. 1:**
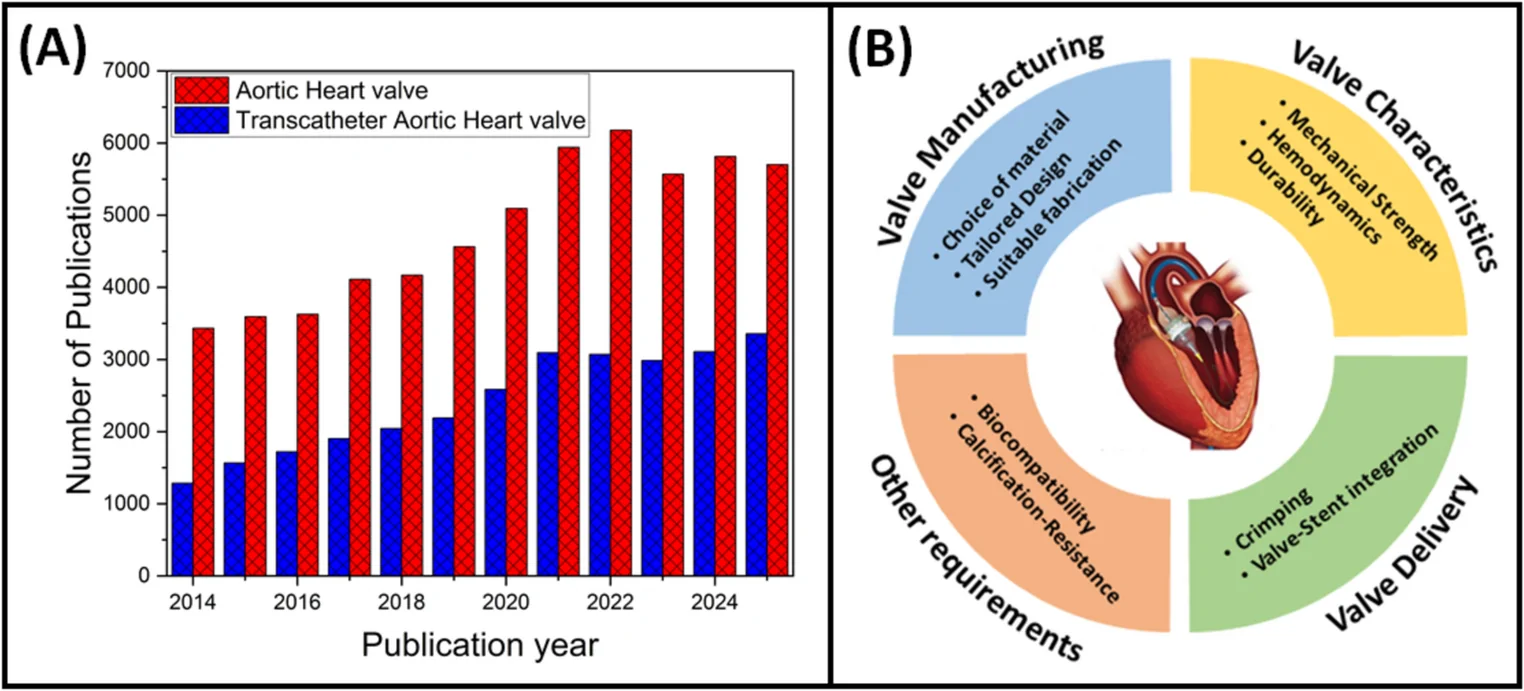
(**A**) Graphical representation of number of publications in past 10 years by searching for “Aortic Heart valve” and “Transcatheter Aortic Heart valve” (Scopus on 14th May 2026). (**B**) Conceptual illustration of various material characteristics required by valves to be suitable for a TAVI approach for implantation

Transcatheter aortic valve implantation (TAVI) is a transcatheter procedure for the implantation of heart valves with a minimally invasive approach, facilitating fast postoperative recovery [11]. The successful implantation of a first heart valve in humans by the TAVI procedure was performed in 2002 [12]. Since then, various companies have developed different types of trans-catheter-based valves, driving significant advancements in the market [13]. DuringTAVI procedure a heart valve mounted on a metallic stent frame is delivered to the aortic annulus through a catheter [14]. While the femoral artery is the most commonly used access route, there are alternative approaches include the subclavian/axillary artery, carotid artery, ascending aorta, left ventricular apex, and inferior vena cava [15]. Once the device is positioned in a desired aortic position, the valve is deployed by either a balloon-expandable or a self-expanding mechanism [16, 17]. TAVI technology has sparked further interest in developing catheter-based heart valves in the last two decades. Advancements in material science coupled with improved fabrication technologies, present a promising opportunity to expand the materials horizon for TAVI procedure [18, 19]. However, the number of research publications on trans-catheter aortic valves technology development is significantly lower compared to surgical aortic valves (Fig. 1A). This could be attributed to the inherent complexity of the material requirements needed to meet the specific demands of the TAVI procedure (Fig. 1B). This review primarily focuses on the design and fabrication of heart valves compatible with the TAVI procedure from a material science perspective. It also compares the materials that are in preclinical and clinical stages, with a focus on further improvements required to enable the widespread adoption of next-generation TAVI valves.

## Materials Challenges in Developing a TAVI Valve

While TAVI technology offers substantial benefits, including minimally invasive treatment and suitability for high-risk patients, manufacturing an aortic valve compatible with the TAVI procedure presents significant challenges due to stringent material requirements before, during and after implantation. Some of the key factors that makes the TAVI valve manufacturing process challenging are discussed below.

### Complex Design and Hemodynamics

The aortic heart valve has an complex structure with three leaflets/cusps in the human cardiovascular system with each leaflet is made of a multi-layered structure comprises of fibrosa, spongiosa and ventricularis [20]. Despite their thinness (300 to 700 μm), native leaflets endure long-term dynamic mechanical stresses, repeated flexing, and rapid pressure fluctuations with every heartbeat. Achieving such a sophisticated biomechanical performance using traditional materials and through the fabrication techniques, such as casting, moulding, coating, electrospinning and 3D printing, presents several challenges [21]. The primary aspect in mimicking the native valve’s hemodynamics is the design/geometry, as it can directly or indirectly influence the stress-distribution across the leaflets and overall durability or valves lifespan [22].

**Fig. 2:**
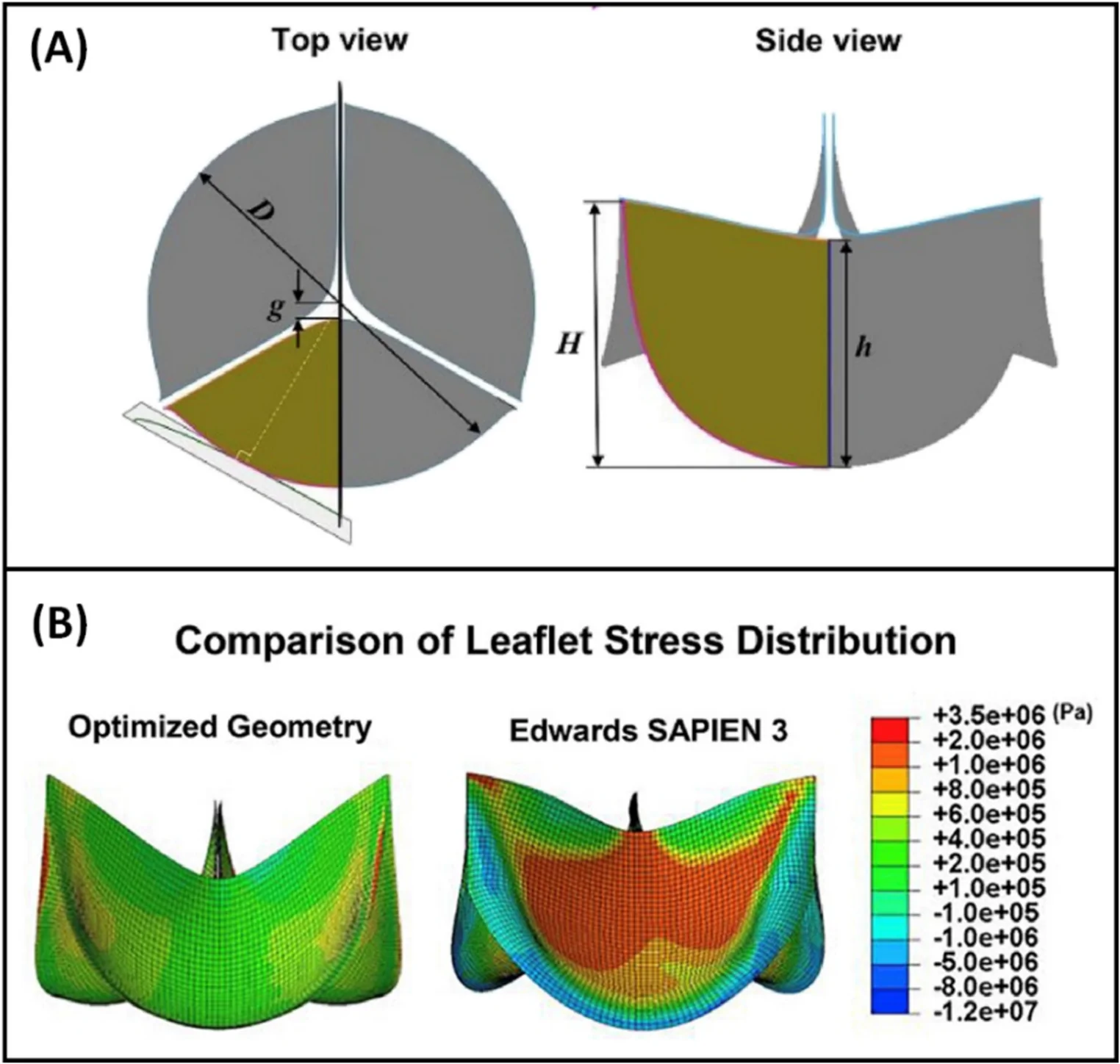
(**A**) Design variables of the TAVI leaflet geometry (D = diameter of the valve; g = gap at the center of the leaflets at the load-free initial configuration, H = valve height, h = leaflet coaptation height) and (**B**) Stress distribution across the leaflets with a slight change in the design. Reproduced with permission [24]. Copyright 2020, Elsevier

The development of a valve requires a design that grants good hemodynamics and minimises the stress distribution across the valve leaflets. Studies using Computational methods like Finite Elemental Analysis (FEA) are able to forecast the stress distribution for a given design or geometry [23]. For instance, Abbas et al. employed ABAQUS Python scripting and MATLAB to develop an automated optimization framework, adjusting parameters such as valve height and leaflet coaptation height to minimize stress distribution across the leaflets [24]. Valve height (H) is the vertical distance between the base of the leaflets and commissures, whereas leaflet coaptation height (h) is the vertical distance between the leaflets base and leaflet contact area between adjacent leaflets during closure (Fig. 2A). Their findings revealed that increasing the ratio of valve height to leaflet coaptation height and increase in curvature of the fixed-boundary edge significantly reduced the maximum stress on leaflets, outperforming the commercially available Edwards SAPIEN 3 valve in terms of stress minimization (Fig. 2B). Similarly, an experimental study conducted on the valves made from poly(L-lactide-co-Ɛ-caprolactone) (PLCL) polymer by focused rotary jet spinning revealed that even slight modifications in valve height, leaflet coaptation height, and curvature significantly altered the valve’s hemodynamic behaviour [25]. Both the simulation and experimental studies highlight a promising opportunity to improve current commercial valve designs by refining geometric parameters, thereby enhancing the long-term durability of TAVI valves through better stress management during valve function. In addition to design variables, the extent of valve expansion during deployment can significantly influence stress distribution across the leaflets and commissures. For instance, a study conducted by Abbasi et al. demonstrated that inadequate expansion of a 23 mm valve to diameters ranging from 18 mm to 23 mm resulted in stress localization in both the commissures and belly regions, resulting in an accelerated tissue degradation [26]. Hence, it is paramount to optimize the valve design to accommodate optimal hemodynamics and distribute the stress across the leaflets when designing the next-generation TAVI valves to achieve the long-term durability.

### Mechanical Properties

**Fig. 3:**
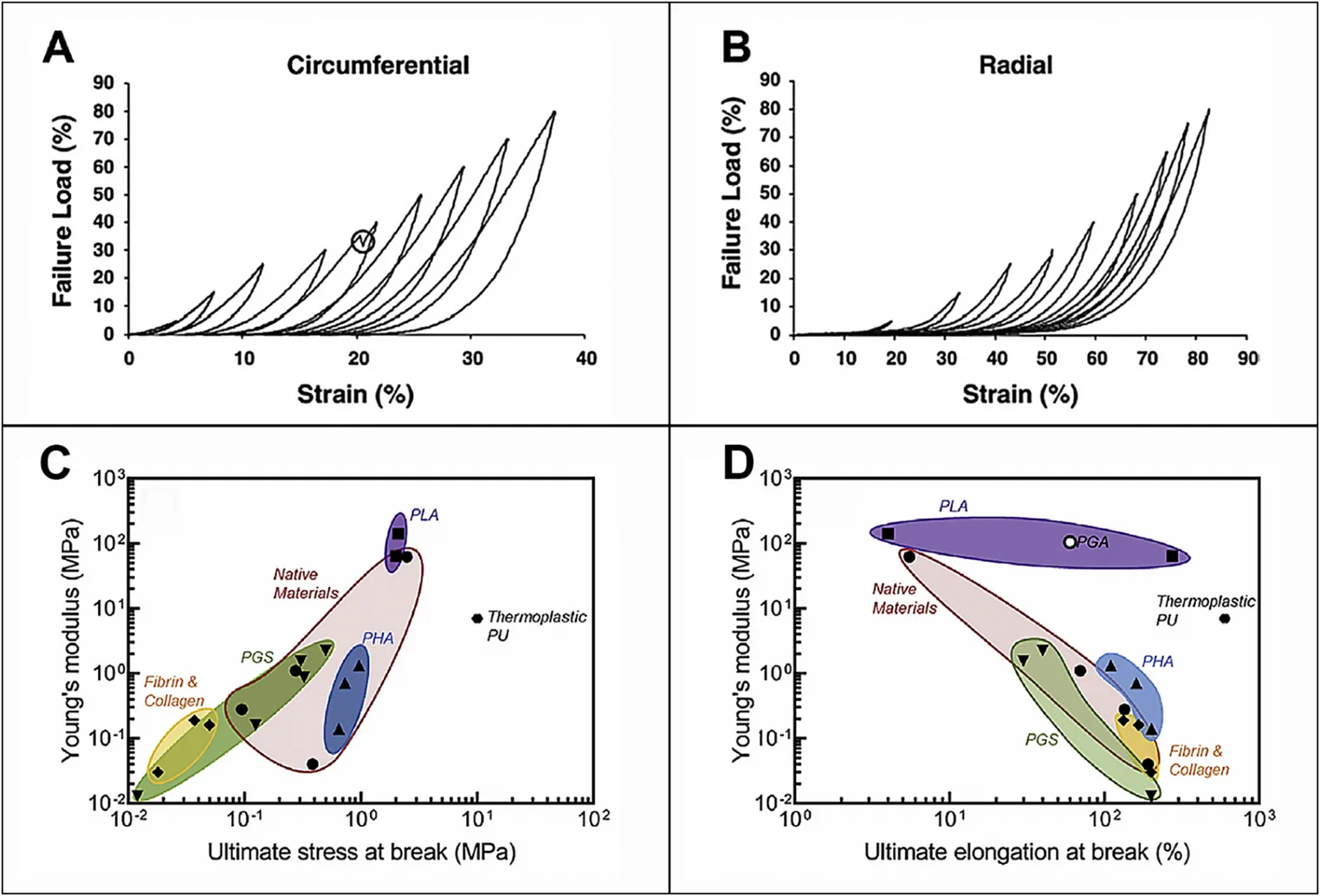
Cyclic load/unload performance of native heart valve in (**A**) circumferential and (**B**) radial directions Reproduced with permission [29]. Copyright 2011, Springer nature. Mechanical properties of various materials when compared to native valves: Young’s modulus versus ultimate stress at break (**C**) and Young’s modulus versus elongation at break (**D**) Reproduced with permission [18]. Copyright 2020, Elsevier

Heart valves experience dynamic mechanical stresses during the unidirectional pumping of blood at each cardiac cycle. On average, a human heart valve undergoes approximately 100,000 cycles per day, translating to about 3 billion cycles over a lifetime [27]. The intricate composition and distribution of structural proteins such as collagen, elastin, and proteoglycans impart anisotropic mechanical properties to the native valve leaflets, and computational models suggests that the heart valve leaflets are stiffer in circumferential direction when compare to radial direction [28]. The experimental investigation by Benam et al. further validate these findings, demonstrating higher strain elongation in radial direction (Fig. 3B) than circumferential direction (Fig. 3A) [29]. This mechanical anisotropy is attributed to the alignment of collagen fibers along the circumferential axis, which is a key factor in the valve’s ability to withstand high cyclic loads [30].

Mechanical anisotropy is crucial for maintaining optimal hemodynamic performance and ensuring valve durability. The energy dissipation characteristics of the native valve, as indicated by hysteresis curves (Fig. 3A and B), show a higher degree of energy loss and irreversibility in the circumferential direction under high strain. Additionally, studies report that the ultimate tensile strength of aortic valve cusps in individuals aged 20 to 50 years ranges from 1.74 ± 0.29 MPa in the circumferential direction to 0.32 ± 0.04 MPa in the radial direction [31]. Engineering this precise anisotropy and strength using traditional materials creates a significant challenge, prompting researchers to explore biomaterials derived from animal tissues [32]. Animal-derived tissues, such as bovine and porcine pericardium, have shown considerable success in mimicking the mechanical anisotropy and hemodynamic performance of native heart valves [33]. However, the majority of them employed glutaraldehyde based cross-linking methods that are prone to calcification, compromising long-term mechanical stability and limits their applicability, particularly for younger patients [34]. Most published studies to date have focused on uniaxial tensile loading and bending properties. However, a more comprehensive investigation over time on viscoelastic properties, creep behaviour, and cyclic loading is necessary to better understand long-term material performance and potential failure modes.

In the past 20 years, advancements in material science and fabrication technologies have led to the development of several tough, flexible materials derived from both natural (collagen, elastin, fibrin, etc.,) and synthetic sources (polyethylene glycol, polylactic acid, polyurethanes, etc.,) that applied to TAVI valve development, could outperform the mechanical properties of the native valves (Fig. 3C and D) [18, 35–37]. For instance, Courtney et al. successfully mimicked the mechanical anisotropy of native heart valves using poly(ester urethane) urea by employing electrospinning techniques [38]. By tuning fibre orientation and varying spinning speeds, they achieved anisotropic mechanical properties that closely resemble those of native valves. However, there is no evidence on the translation of these innovations into a functional heart valve, with the correct shape, hemodynamics and durability for TAVI applications. There is a significant scope and need to integrate mechanical anisotropy into complex heart valve geometries to better mimic the native valve function. Advanced fabrication tools such as 3D printing, melt electro-writing, rotary jet spinning, and other microfabrication techniques offer promising pathways to achieve this while maintaining performance and ensuring long-term durability. For instance, Vernon et al. used melt electrowriting (MEW) to fabricate a tissue scaffold with tailored fibre orientations to mimic the collagen distribution and orientation found in native valves [39]. Notably, the scaffold exhibits similar yield strain, hysteresis, and relaxation behaviour to porcine heart valves, demonstrating a significant step toward achieving structural and mechanical biomimicry in heart valve engineering. However, the translational aspects such as making it into a valve shape and securing it on stent without compromising the structural and mechanical anisotropy remains to be investigated.

### Calcification Resistance

Calcification is characterized as the progressive accumulation of calcium ions on the valve leaflets leading to their stiffening, immobilization and subsequent structural failure. The underlying mechanism for calcification in natural heart valves is not fully understood to date as it depends on multiple factors including genetics, induced stresses, aging and inflammation [17, 40]. On the other hand, the calcification mechanism in bio-prosthetic valves, primarily derived from glutaraldehyde-fixed bovine and porcine pericardium, have been extensively investigated [18]. Although, these bio-prosthetic valves are widely employed in clinical settings, they are prone to long-term calcification, limiting their utility in younger or more active patients. Efforts to mitigate calcification in bio-prosthetic valves have largely focused on alternative crosslinking strategies such as replacing glutaraldehyde, a known promoter of calcification. Non-glutaraldehyde crosslinking agents such as epoxy compounds, have demonstrated significant reductions in calcification in in-vivo rat subdermal models compared to glutaraldehyde-treated tissue [41]. Nevertheless, despite promising advances, complete prevention of calcification in bio-prosthetic valves remains elusive, as calcification is influenced not only by the chemical composition of the material but also by its surface properties, morphology, and microstructural characteristics. Even chemically inert materials can exhibit calcification due to ion anchorage facilitated by surface roughness or porous structures, which serve as nucleation sites for mineral deposition [42].

Synthetic polymer heart valves offer distinct advantages in terms of calcification resistance due to their non-biological composition. For instance, valves fabricated from silicone-based polyurethane (CarboSil^®^) demonstrated minimal to no calcification compared to glutaraldehyde-fixed bovine pericardium valves when implanted in rats over a six-week period [43]. This highlights the potential of polymeric valves in overcoming the calcification issues associated with bio-prosthetic valves. Beyond material composition, surface modifications have shown significant promise in controlling mineral deposition. For example, bovine pericardium coated with silk fibroin (SF) displayed no signs of calcification during in-vitro calcification assays [44]. In another in-vivo study, electro spun SF/PU (polyurethane) patches implanted in rats did not show any sign of calcification over a period of 3 months [45]. Similarly, scaffold made from polycaprolactone (PCL) and SF using showed calcification levels much lower than clinically used porcine pericardium [46].

Despite these advances, calcification remains a major challenge, hindering the broader adoption of bio-prosthetic valves in younger and more diverse patient populations. Using the biocompatible tailor-made polymers that are inert to biological cues can be an attractive alternative to biological tissue valves. Furthermore, surface modification through advanced polymer chemistry offers a promising pathway to enhance the performance and longevity of current bio-prosthetic valves.

### Durability

Durability is one of the key factors that determines whether the valve outperforms the patient’s lifespan or needs another surgical intervention. According to ISO 5840 standards, heart valves must withstand a minimum of 200 million cycles (equivalent to 5 years) to be considered viable for clinical use [47]. Currently, bioprosthetic valves are the most widely utilized in TAVI procedures, with clinical data supporting reliable performance for up to 5 to 8 years post-implantation [48, 49]. While this level of durability may be sufficient for elderly or high-risk patients, it remains inadequate for younger patient populations, where extended valve longevity is critical. Consequently, improving the durability of bio-prosthetic valves is a pressing objective in extending TAVI treatment to younger patients. Despite numerous studies aimed at enhancing bio-prosthetic valve performance, clinical data extending beyond 8 years remain limited [48, 49].

On the other hand, polymeric valves have demonstrated superior durability when tested in-vitro, with some studies reporting survival of up to 1.3 billion cycles, equivalent to a 30-year lifespan [47]. Although polymeric valves showed significantly higher in-vitro durability, further optimization of the valve to fit the TAVI procedure and their translational outcomes are very limited. For instance, several valves made from polycarbonate urethanes showed the durability of 600 to 1000 million cycles in accelerated in-vitro studies but their translation towards a TAVI procedure has not been reported to date [50]. In addition to intrinsic material properties, external factors, such as crimping and valve-stent integration, significantly influence valve durability. Crimping, in particular, imposes substantial mechanical stress on the valve leaflets, affecting long-term performance. For example, studies on pericardial valves have shown that crimping to reduced diameters can lead to nearly a 50% decrease in tensile strength, directly compromising durability [51]. Unfortunately, many investigations into durable polymeric valves do not include critical parameters, such as crimping effects and stent integration processes, which are essential for understanding their feasibility for a TAVI procedure.

### Crimping

**Fig. 4:**
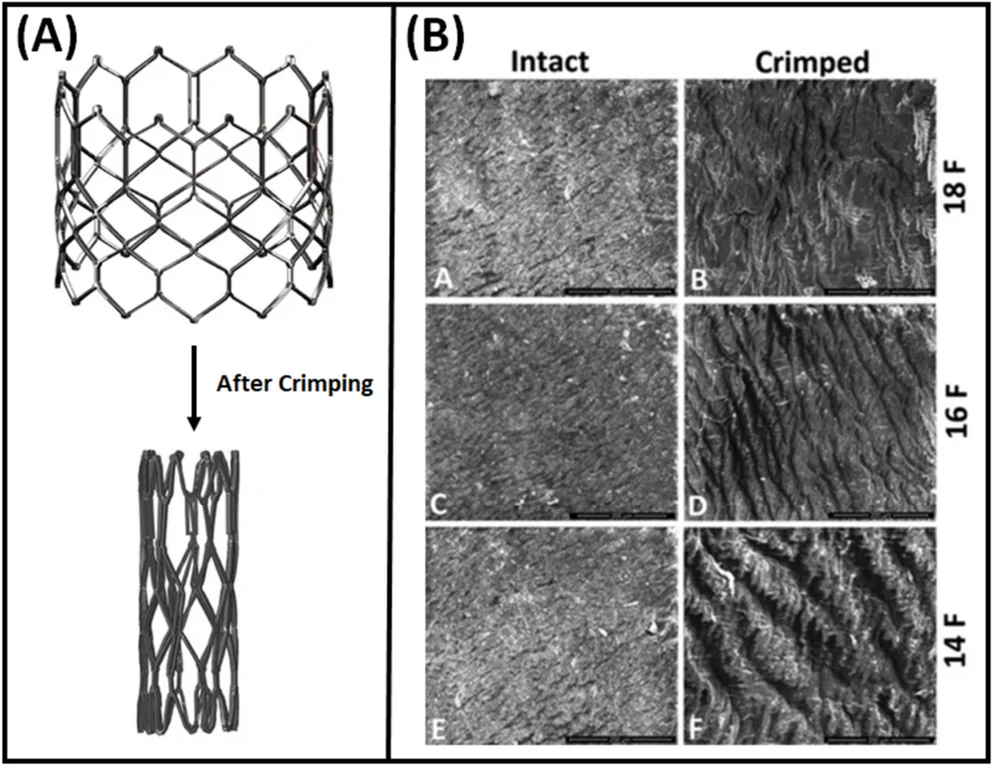
(**A**) Pictorial representation of stent crimping to smaller size. Reproduced with permission [56]. Copyright 2021, Wiley. (**B**) Scanning electron microscopy images show intact (**A**, **C**, and **E**) and crimped (**B**, **D**, and **F**) states of three different pericardial leaflets at 18 F, 16 F, and 14 F, respectively. Reproduced with permission [54]. Copyright 2014, Elsevier

Crimping is an essential pre-surgical step in the TAVI procedure, where the valve mounted on a metallic stent is reduced to a low diameter (typically from 23 mm to 5 mm) to facilitate the catheter-based delivery (Fig. 4A) [52]. Clinical studies indicate that during the TAVI procedure the valve remains in crimped state for approximately 20 to 60 min. While necessary for procedural success, this temporary but intense mechanical compression can induce substantial stress on the valve leaflets, potentially accelerating structural degeneration or failure [53]. The adverse effects of crimping have been extensively documented, particularly in bio-prosthetic valves. Studies have shown that the high mechanical stresses imposed during crimping can cause irreversible damage to pericardial leaflets. For instance, crimped pericardial tissue exhibited significant structural deterioration, as evidenced by surface defects and micro-scale cracking observed through scanning electron microscopy (SEM) (Fig. 4B) [54]. Moreover, the degree of leaflet wrinkling and cracking increases as the crimped diameter decreases, further compromising valve integrity. Reducing crimping duration and avoiding extreme diameter reductions have been proposed as strategies to mitigate structural damage in biological valves [54]. On the other hand, aortic valves made from the silicone-polycarbonate-urethane showed no significant surface damage with crimping demonstrating the potential of polymeric valves in overcoming the crimping challenges [55].

In parallel, polymeric valves have emerged as promising alternatives due to their superior in-vitro durability. Several polymeric valve designs have demonstrated excellent longevity, surviving millions of cycles under accelerated wear conditions [5]. However, despite their potential, a critical gap in the translational pathway still remains: many of these studies lack comprehensive crimping analyses, which are crucial to assess the valve’s suitability for real-world TAVI applications. Without crimping data, it is difficult to predict how polymeric valves will perform under the mechanical stresses inherent to TAVI procedures. Future research must address this gap by systematically evaluating the crimping performance of polymeric valves. Understanding the interplay between crimping-induced stresses, leaflet integrity, and long-term durability will be essential in advancing polymeric valve technologies from bench to bedside. By optimizing valve materials and design for crimping resilience, it may be possible to overcome a major limitation of current bio-prosthetic valves and pave the way for next-generation TAVI solutions. However, the most available clinical data on polymeric valves is limited to several months and patients are still under anticoagulation. Therefore, its potential to replace the biological valves has to be demonstrated by long-term clinical studies to propose them for younger and lower-risk patient populations.

### Valve Stent Integration

Stent composition and design plays an important role in TAVI procedures, governing not only the delivery mechanism but also valve hemodynamics and long-term durability. Despite its importance, this aspect is often overlooked in the current literature. The choice of stent material and design directly determines the deployment mechanism, whether through self-expansion or balloon expansion [57]. For instance, stainless steel and cobalt-chromium stents typically require balloon expansion, whereas nitinol-based stents are deployed via self-expansion [58]. In addition, rhenium based stents delivered through balloon expansion are 2 to 3 times stronger than traditional stents offering improved radiographic visibility and reduced delivery system size (i.e. Siegel TAVR) [59]. Integrating the valve with the stent is a crucial manufacturing step that ensures structural integrity and optimal hemodynamic performance. Most bio-prosthetic valves, made from animal tissues are secured to the stent frame through precise suturing to maintain long-term durability [60]. However, suturing demands a high degree of craftsmanship and cannot be automated, thereby increasing manufacturing costs. There has been some progress in reducing the number of sutures to minimize tissue damage during integration [61]. In addition to suturing, sealing cuffs or skirts made from polyethylene terephthalate (PET) are incorporated in commercial devices such as the SAPIEN XT (Edwards Lifesciences) and DurAVR™ (ANTERIS Technologies) to reduce paravalvular leakage, a common complication in the TAVI procedure [57]. Furthermore, DurAVR™ valve is engineered to mimic the native aortic valve geometry from a single piece bovine pericardial tissue [62]. The single piece tissue valve integrated on the stent by suturing showed superior hemodynamic performance when compared to current devices such as the SAPIEN 3, Evolut PRO, Navitor, and ACURATE neo 2 [63].

An emerging alternative to conventional suturing is the direct fabrication of valves onto stent frames, a suture-less approach that has the potential to eliminate suturing-related errors and minimize the need for additional sealing skirts. However, this approach requires a careful synergy between stent material and polymeric leaflet components to achieve reliable adhesion, which is a challenge in many existing designs. One innovative solution involves the use of adhesion promoters to enhance stent-leaflet bonding. For instance, 3-aminopropyltriethoxysilane (γ-APS) was employed as a coupling agent to bond a hybrid polymer composed of polyhedral oligomeric silsesquioxane (POSS) and poly (carbonate-urea) urethane (PCU) to a nitinol stent [64]. This approach increased bond strength by a factor of three, resulting in a suture-less valve that endured over 400 million cycles without significant mechanical drop [65]. Despite these promising results, further investigations into suture-less valve technologies remain limited. Notably, devices such as the Foldax Tria and TRISKELE valves have adopted suture-less designs for polymeric TAVI valves [66–68]. However, detailed descriptions of their manufacturing processes remain undisclosed, likely due to proprietary considerations. Moving forward, addressing the integration challenges between stent and valve materials, as well as ensuring robust attachment under crimping and cyclic loading conditions, will be critical for advancing suture-less valve technologies.

## Current TAVI Valves from a Materials Perspective

Although several materials have been explored for fabricating aortic valves, only a limited number of successful TAVI devices have been translated into clinical practice, owing to the inherent complexities discussed in the preceding sections. The combined efforts from scientists, engineers, interventional cardiologists and surgeons have resulted in a handful of TAVI valves from various sources which are currently in preclinical and clinical trials. From the materials point of view, they can be classified as biological and polymeric TAVI valves as discussed below.

### Biological TAVI Valves

**Fig. 5:**
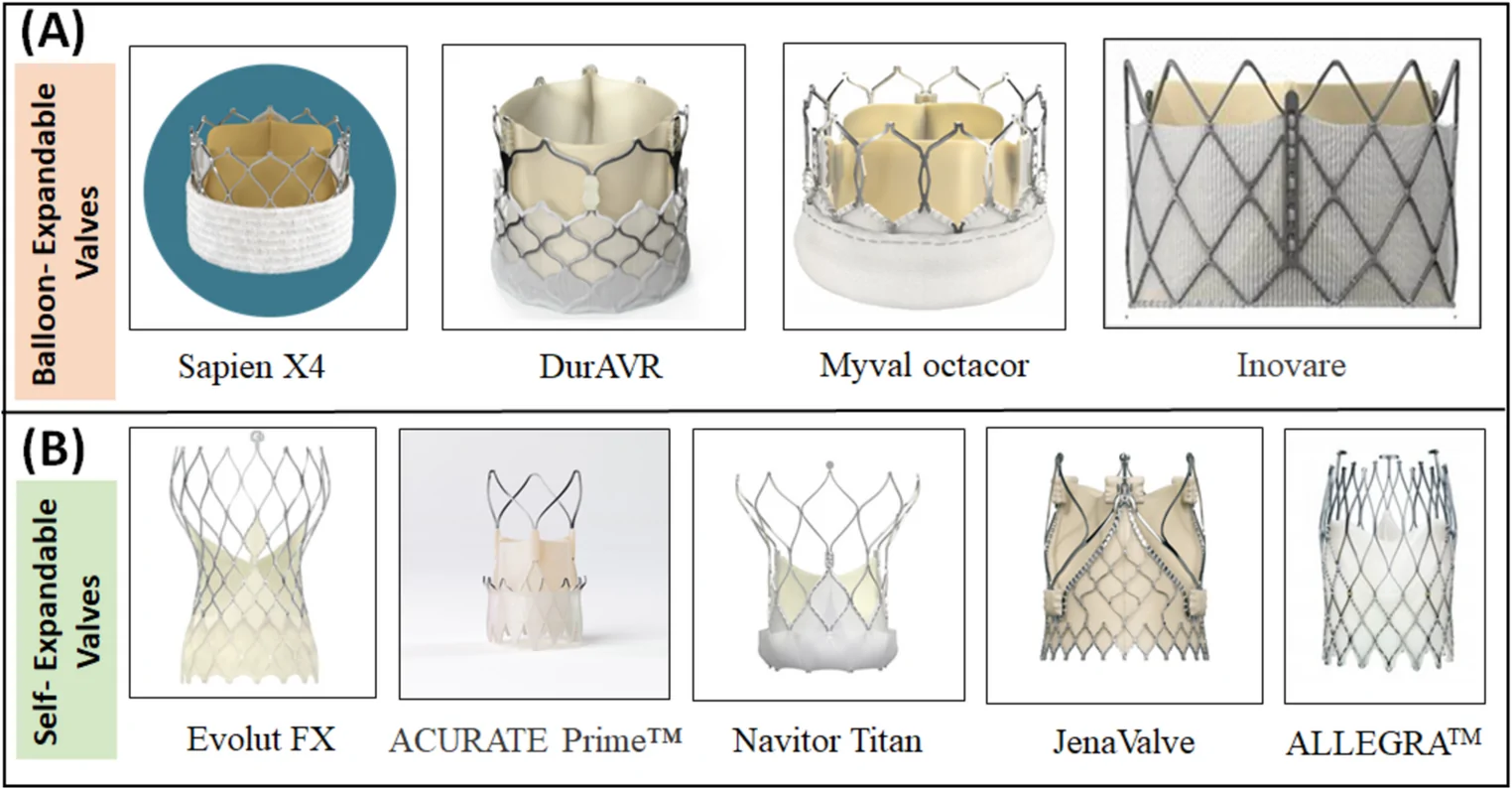
Optical images of the (**A**) Balloon-expandable and (**B**) Self-expandable biological TAVI devices. Reproduced with permission [57]. Copyright 2023, Taylor & Francis

Biological TAVI valves, are predominantly derived from animal tissues such as bovine or porcine pericardium (Fig. 5). The first successful human implantation of a TAVI valve utilized a tri-leaflet valve made from bovine pericardium mounted on a stainless-steel frame and delivered via a balloon-expandable catheter through the femoral artery [69]. Despite the initial success, follow-up studies conducted over two years revealed mild to severe aortic regurgitation due to paravalvular leaks. Moreover, its in-vitro durability testing at the time showed a lifespan of only 100 million cycles, restricting its use to high-risk, older patients [69, 70].

Since then, biological TAVI valves have seen significant changes in terms of both material improvements and stent design making it a preferable therapeutic option for aortic stenosis treatment [71]. Among them, SAPIEN 3, SAPIEN 3 Ultra, and SAPIEN 3 Ultra RESILIA are well known TAVI valves developed by Edwards Life Sciences. These valves, derived from bovine pericardium, are attached to balloon-expandable stents with Polyethylene terephthalate (PET) skirts designed to minimize paravalvular leakage (PVL) [57]. The latest version from the Edwards Life Sciences is the SAPIEN X4, incorporates RESILIA Tissue, a next-generation bovine pericardium treated with enhanced anti-calcification technology to improve long-term durability [72, 73]. The tri-leaflet RESILIA Tissue is attached to a balloon-expandable chromium-cobalt frame with low frame height and larger cells for easier coronary access, while an improved PET skirt further reduces paravalvular leaks. The SAPIEN X4 is still under clinical evaluation and is not commercially available.

Myval Octacor is another TAVI device made from bovine pericardium, which is attached to a balloon-expandable nickel-cobalt alloy frame [74]. Anti-calcification is achieved through proprietary AntiCa-Merils technology, and PVL is mitigated using PET skirting. Similarly, DurAVR is another device made from bovine pericardium attached to a short balloon-expandable chromium-cobalt frame by suturing [75]. Like Sapien X4 and Myval Octacor, a PET skirt is used to reduce the paravalvular leaks, and coronary access is facilitated by the large open cells of the frame. It is made from a single piece biological tissue treated with proprietary ADAPT tissue engineering process to get the desired strength and elasticity with anti-calcification properties [76]. Inovare, is yet another bovine pericardium based TAVI device, commercially available in Brazil [77]. It is made from a single sheet of bovine pericardium on a balloon-expandable chromium cobalt frame, but the crosslinking mechanism and anti-calcification technology remain undisclosed. The use of single sheet minimizes the number of pericardium sutures, which is a potential culprit compromising the valve durability.

Medtronic (Minneapolis, MN) has pioneered self-expandable biological TAVI valves with its Corevalve, Evolut R, Evolut Pro, Evolut Pro + and Evolut FX devices [78]. All of these are made from the porcine pericardium sutured on to a nitinol frame. The latest iterations have an external porcine pericardial wrap designed to reduce PVL, addressing limitations observed in earlier models [79]. Boston Scientific’s ACURATE Prime and ACURATE neo2 valves employ porcine pericardium on self-expanding nitinol frames, incorporating inner and outer skirts made of porcine tissue to further minimize PVL [80, 81]. The ACURATE neo2 was discontinued after it failed to demonstrate superior performance when compared to commercially available valves like Sapien or Evolut [82]. Other noteworthy devices include Navitor Titan, TaurusElite, SinoCrown, ALLEGRA™, and JenaValve, all of which use bovine or porcine tissues attached to self-expanding metal stents with external tissue wraps to reduce PVL [57]. Among them Jena valve is used for aortic regurgitation (and therefore is designed to be implanted in a non-calcified aortic tissue) and both TaurusElite and SinoCrown are currently only available in China. In summary, despite the widespread use of animal tissues in biological TAVI valves, intricate details regarding tissue treatment processes and anti-calcification crosslinking technologies are often proprietary technology and not publicly disclosed [32]. While most biological valves rely on sutures to attach the valve to the stent frame, this raises concerns about long-term durability and manufacturing complexity. Moreover, suturing demands skilled craftsmanship and cannot be easily automated, contributing to increased production costs. Although there have been efforts to reduce the number of sutures to mitigate tissue damage, further innovation is required [61]. Developing suture-free integration methods and bio-inert surface coatings to enhance calcification resistance and durability represents a promising direction for future research.

### Polymeric TAVI Valves

**Fig. 6:**
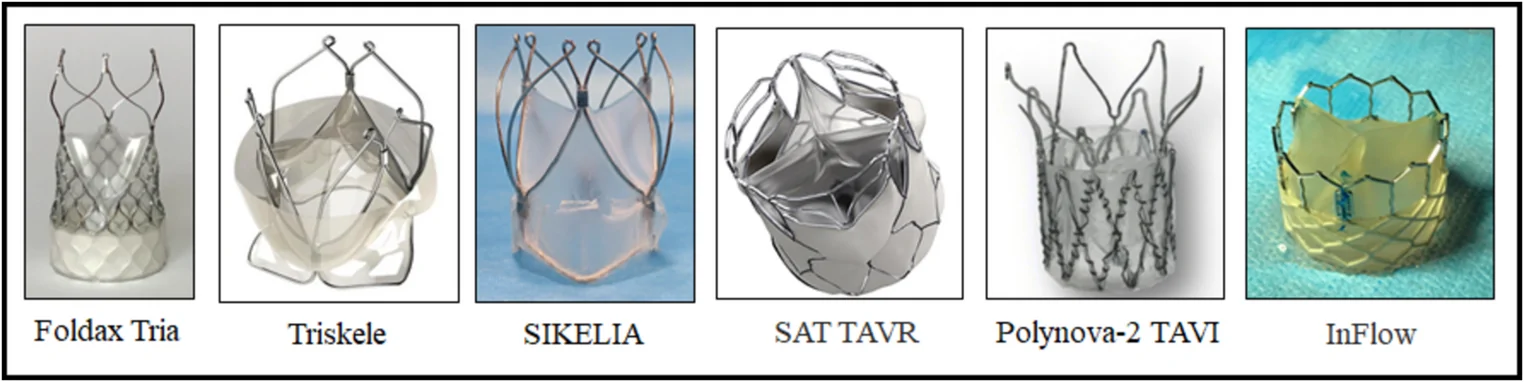
Optical images of the polymeric TAVI devices that are at various stages of development. Reproduced with permission [84]. Copyright 2024, Elsevier

Polymeric materials are widely investigated and utilized in the medical industry because they offer precise tunability in physiochemical properties by controlling the molecular architecture of the materials [83]. Polymeric valves offer great advantages in terms of calcification resistance and in-vitro durability when compared to biological valves [84, 85]. Several polymers have been explored in the development of TAVI valves ranging from polysiloxanes, polyurethanes, styrene triblock copolymers [47, 86]. However, only a few of them have been translated successfully to pre-clinical and clinical testing stages (Fig. 6).

Polynova-2 TAVI is one prominent example developed by Stony Brook University and Polynova Cardiovascular Inc. based on their previous Polynova surgical valve. The valve leaflets are fabricated from a novel polyisobutylene-based copolymer using vacuum compression molding at high temperatures (220 °C) and pressures (4 tons) [87]. The valve leaflets are attached to a nitinol stent frame by suturing, which showed excellent hemodynamics and resistance to calcification. However, as the reported durability of 400 million cycles is not tested on the stent-integrated valve, raising concerns about its clinical feasibility [88]. The Polynova-2 TAVI is still under preclinical/bench developmental stage.

At University College London (UCL), researchers developed a TAVI device called TRISKELE using a hybrid polymer composed of polyhedral oligomeric silsesquioxane (POSS) and poly(carbonate-urea) urethane (PCU). The valve leaflets and sealing cuffs are manufactured by robotic dip coating of 18 wt% polymer solution. While the device demonstrated superior hemodynamics compared to biological valves such as SAPIEN XT (Edwards Lifesciences) and CoreValve (Medtronic) in sheep models, details regarding the attachment mechanism remain undisclosed, likely due to intellectual property protection [66]. The enrolment for first-in-human trials were anticipated in 2023, however no updates have been reported.

Foldax has developed a polymeric TAVI valve called Foldax Tria. They employed a silicone-based polyurethane urea (SiPUU) called LifePolymer to make the valve leaflets because of its bio-stability, mechanical strength and creep resistance [67, 89]. The LifePolymer is seamlessly integrated into the self-expanding nitinol frame and successfully tested in sheep model. Recently, Stanfield et al. reported comprehensive preclinical results on this device and it successfully completed 200 million cycles of fatigue testing without fracture or deformation [90]. In a 90-day ovine study involving nine animals (six successful implants), the valve demonstrated stable hemodynamics, no hemolysis, absence of paravalvular leak at implantation, and critically, no calcification at explant. In addition, Foldax LifePolymer showed encouraging clinical results by demonstrating no calcification and no structural valve deterioration even after one year, when surgically implanted in mitral position [91].

Another noteworthy development is the InFlow valve, a hybrid polymeric TAVI device created by a team in Poland [92]. The tri-leaflet design incorporates polyurethane-co-carbonate (PU) and polycarbonate-co-silicone (PUS) attached to a cobalt-chrome frame, though the exact attachment method remains undisclosed. Post-implantation evaluation in sheep revealed thrombi and scanty calcification in some cases after 180 days. The InFlow polymeric valve is in preclinical stage with in-vitro durability exceeding 20 years, surpassing the traditional biological valves, making it a potential candidate for younger patients. Shanghai Yixin Med (China) implanted the first synthetic polymer-based TAVI valve called SIKELIA™ in 80-year-old human [93]. Followed by this it is implanted in 12 patients and the follow-up study after 12 months showed favourable hemodynamics with no side effects [94]. The leaflets in their device is made from a polyurethane based nanocomposite called BioDura^®^ and attached to a nickel-titanium stent without sutures, which can facilitate scalable production. However, the exact manufacturing methods remains undisclosed. In addition to scalable production, polymeric valves can offer superior in-vitro durability with precise quality control and lower production costs which is a major challenge in the current bio-prosthetic heart valves in the market [95].

In addition to materials that have reached the pre-clinical and clinical trials, there are some noteworthy studies that used innovative approaches to make the TAVI valves. For instance, Guo et al. developed a TAVI valve by ultraviolet light crosslinking of Poly (ethylene glycol) diacrylate (PEGDA) hydrogel on a polyethylene terephthalate/polyamide6 (PET-PA6) fabric on a nitinol frame [96]. This study showed excellent hemodynamics with anti-fouling properties; however, there is no details on the durability on the device to demonstrate the future potential. In other study, Motta et al. used focused rotary jet spinning to mimic the collagen fibre architecture using a hybrid polymer made from poly-lactic acid (PLA) and polycaprolactone (PCL) [25]. The fibrous leaflets were sutured onto a nitinol stent frame and implanted in rats for short-term in-vivo evaluation. The authors reported no adverse reactions in the rats and observed stable hemodynamic performance. However, the in-vivo assessment was limited to a duration of 1 h, leaving the long-term durability uncertain.

Shifting away from conventional integration of valves to metal stents Chen et al. developed a new fabrication method to manufacture a Polyetheretherketone (PEEK) based heart valve with a continuous stent-leaflet interface [97]. Rather than manually stitching the leaflet which induces localized stress concentrations, this study introduced a modified vacuum forming method to simultaneously shape and thermally weld 50 μm thick semi-crystalline PEEK leaflets directly onto the composite frame. While this method enables rapid manufacturing with improved stent-leaflet bonding, further optimisation to overcome the early signs of plastic deformation and durability remain to be explored.

Although many polymeric valves have exhibited impressive in-vitro durability in accelerated wear tests (AWT), only few have progressed to preclinical (Polynova-2 TAVI, TRISKELE, InFlow, ) and clinical trials (Foldax Tria, SIKELIA™ ) [85]. An interesting trend noticed is that all the polymeric valves that have reached the pre-clinical and clinical trials employed suture-less technology for valve-stent integration. This suggests that future research should focus on developing biocompatible adhesives that can facilitate stronger valve-stent attachment and seamless integration, thereby advancing the commercial viability of polymeric TAVI valves.

## Conclusions and Future Perspectives

In this review, we discussed the recent advancements in the treatment of Aortic Stenosis through minimally invasive TAVI procedure, focusing from the material science perspective. Significant progress has been made in developing heart valves from both natural and synthetic materials that are compatible with TAVI procedure. It is evident that, biological valves derived from animal tissues are widely commercialized, and their use is still more common compared to synthetic valves. However, their limited calcification resistance and longer-term durability hinder broader application, particularly for younger patients. There is significant scope in adopting new technologies, such as surface coatings to enhance calcification resistance and bio-friendly crosslinking methods to improve valve durability.

On the other hand, polymeric valves offer superior calcification resistance and durability when tested in in-vitro. Despite these advantages, most polymeric valves have not seen clinical translation due to challenges such as crimping stability and valve-stent integration. To date, only three polymeric TAVI devices have reached the clinical trial phase, highlighting the significant gap in integrating valve manufacturing with collapsible stent design. Future research should focus on innovative manufacturing approaches that enable seamless valve-stent integration. Additionally, the development of biocompatible adhesives to enhance the synergy between valve and stent materials could accelerate the commercialization of synthetic TAVI valves.

Overall, the advances in material science fundamentals, engineering design and advanced manufacturing methods have resulted in a handful of TAVI devices. There is an active, transition of TAVI valve leaflet materials from exclusively glutaraldehyde‑fixed bovine/porcine pericardium towards engineered polymeric and hybrid platforms designed to improve durability, reduce calcification and thrombosis, and enable thinner leaflets for better hemodynamics. However, a detailed analysis on unknown in-vivo thrombogenicity of polymeric valves remain to be addressed. While polymeric valves face significant challenge for clinical adoption due to high safety and performance standards set by the biological valves, recent clinical evidence on polymeric valve (from Foldax, Inc.) showed positive outcomes. This suggests that future focus on polyurethane-based hybrids, coupling with robust stent integration mechanism and scalable production strategies, can have a huge potential to expand its use in younger patients and meeting future demand.

## Abbreviations

Α: 3-aminopropyltriethoxysilane
AS: aortic stenosis
AWT: accelerated wear tests
CFD: computational fluid dynamics
FEA: :finite elemental analysis
PCL: polycaprolactone
PCU: poly (carbonate-urea) urethane
PEGDA: poly (ethylene glycol) diacrylate
PET: polyethylene terephthalate
PET-PA6: polyethylene terephthalate/polyamide6
PLA: poly-lactic acid
PLCL: poly(L-lactide-co-Ɛ-caprolactone)
POSS: polyhedral oligomeric silsesquioxane (POSS)
PU: polyurethane-co-carbonate
PVL: paravalvular leakage (PVL)
SEM: scanning electron microscopy
SF: silk fibroin
SiPUU: silicone-based polyurethane urea
TAVI: trans-catheter aortic valve implantation
UCL: University College London
